# A Protein Editing Method to Modify Proteins in Live Mammalian Cells

**DOI:** 10.1002/cpz1.70443

**Published:** 2026-08-21

**Authors:** Jenna N. Beyer, Kaitlyn Toy, George M. Burslem

**Affiliations:** ^1^ Department of Biochemistry and Biophysics, Perelman School of Medicine University of Pennsylvania Pennsylvania PA USA; ^2^ Department of Cancer Biology, Perelman School of Medicine University of Pennsylvania Pennsylvania PA USA; ^3^ Epigenetics Institute, Perelman School of Medicine University of Pennsylvania Pennsylvania PA USA

**Keywords:** biology, chemical biology, click chemistry, genome editing, noncanonical amino acid, protein Editing

## Abstract

Protein editing, as mediated by protein trans‐splicing, offers the ability to manipulate, label, and modify proteins in vitro and in live mammalian cells. This ability to observe and control proteins with this high resolution is a burgeoning area of interest within the chemical biology field. In this protocol, we outline the general approach for setting up a cellular protein editing experiment, emphasizing the planning process for a successful protein editing experiment. Additionally, we include methods to install and subsequently label a click chemistry handle *p*‐azido‐phenylalanine, a non‐canonical amino acid (ncAA) within the intein donor, such that this ncAA and any label can be edited into a protein in live mammalian cells. Overall, we anticipate that this protocol should serve as a generalizable roadmap for planning and carrying out a protein editing experiment. © 2026 The Author(s). *Current Protocols* published by Wiley Periodicals LLC.

**Basic Protocol 1**: Expression of intein donor containing pAzF

**Basic Protocol 2**: Purification and labeling of recombinant intein donor

**Basic Protocol 3**: Preparation of mammalian cells for editing experiments

**Basic Protocol 4**: Protein editing via electroporation

## INTRODUCTION

Protein editing, as mediated by protein trans‐splicing, offers the ability to manipulate, label, and modify proteins in vitro and in live mammalian cells with residue‐level precision and minute‐scale temporal resolution. This ability to observe and control proteins with this high resolution is a burgeoning area of interest within the chemical biology field (Toy et al., 2026). Recent works from our group and the Muir group have established this protein editing technology as a next‐generation tool for manipulating and studying proteins with myriad potential applications (Beyer et al., 2025; Hua et al., 2025). This protein editing approach enables precise experimental control at the residue level of proteins, which can readily be extended to endogenous proteins. Further, the approach enables temporal control over experiments, where proteins can be edited in less than 10 minutes. To date, this technology has been used to visualize and isolate endogenous proteins without the use of antibodies, investigate half‐lives of proteins, and other applications. Notably, the resulting edited proteins remain functional when tested for key attributes, suggesting that protein editing is a minimally disruptive technique.

**Figure 1:**
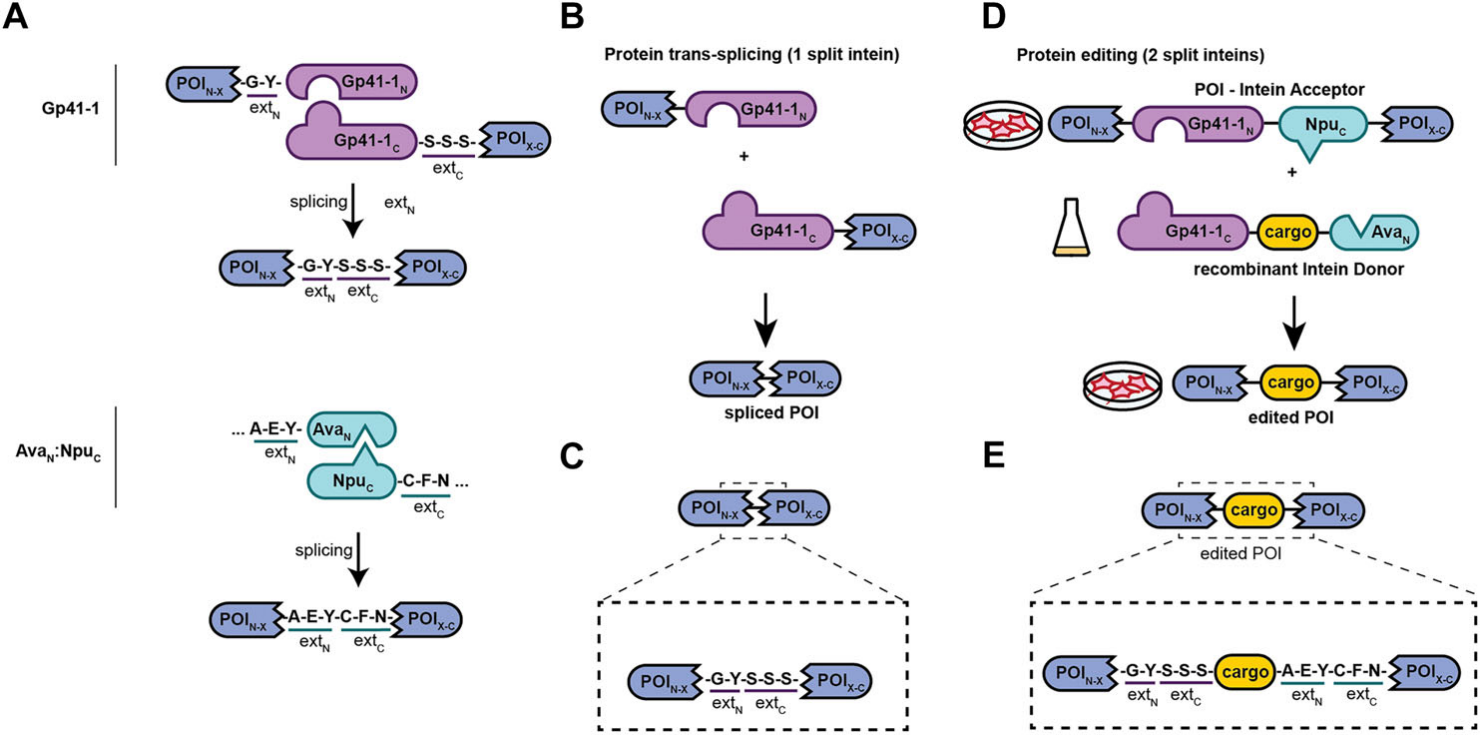
Split inteins as adaptable tools for modifying a protein of interest (POI) in mammalian cells. **(A)** Split inteins possess flanking extein residues (abbreviated extN and extC) that impact splicing efficiency. The native extein residues for Gp41‐1 and AvaN:NpuC are shown. **(B)** Single split intein pair–mediated splicing can be used to modify N‐ or C‐termini of a POI. **(C)** The resulting spliced POI from **(B)** contains the extein residues following splicing. **(D)** Protein editing, using 2 pairs of split inteins, uses a POI tagged with “intein acceptor” in mammalian cells and the corresponding “intein donor” produced in *E. coli*. Delivery of the intein donor to the mammalian cells yields an edited POI. **(E)** The edited POI from **(D)** contains the extein residues from both split inteins, situated around the “cargo” edited into the POI.

Protein editing relies upon split inteins to carry out the protein trans‐splicing required for editing (Fig. 1a). Split inteins are corresponding *N‐* and *C‐*protein domains that can carry out protein trans‐splicing, or the joining of two separate peptides together via a native peptide bond (Anastassov et al., 2024; Shah & Muir, 2013; Volkmann & Mootz, 2013). Splicing often requires extein residues that flank the splicing site, where changes to these residues can have implications for splicing efficacy. Numerous previous studies have used a single split intein pair to splice peptides onto proteins at either terminus (Fig. 1b and 1c), including inside live mammalian cells (Burton et al., 2020; David et al., 2015; Lee & Muir, 2024).

When arranged onto two opposing constructs, two pairs of split inteins can carry out protein editing, where a peptide from one construct (“intein donor”) is inserted into the site demarcated by the other construct (the “intein acceptor”). This configuration has also been termed “tandem protein trans‐splicing” and has been applied in vitro (Shi & Muir, 2005) and with synthetic peptides in mammalian cells (Khoo et al., 2020). In the protein editing workflow described herein, the intein acceptor–tagged target protein is expressed in mammalian cells, while the intein donor is expressed recombinantly in *E. coli*. The intein donor is then purified, labeled (if applicable), and delivered into mammalian cells expressing the intein acceptor. With all intein constructs inside the cells, editing can then occur (Fig. 1d and 1e) (Beyer et al., 2025).

**Figure 2:**
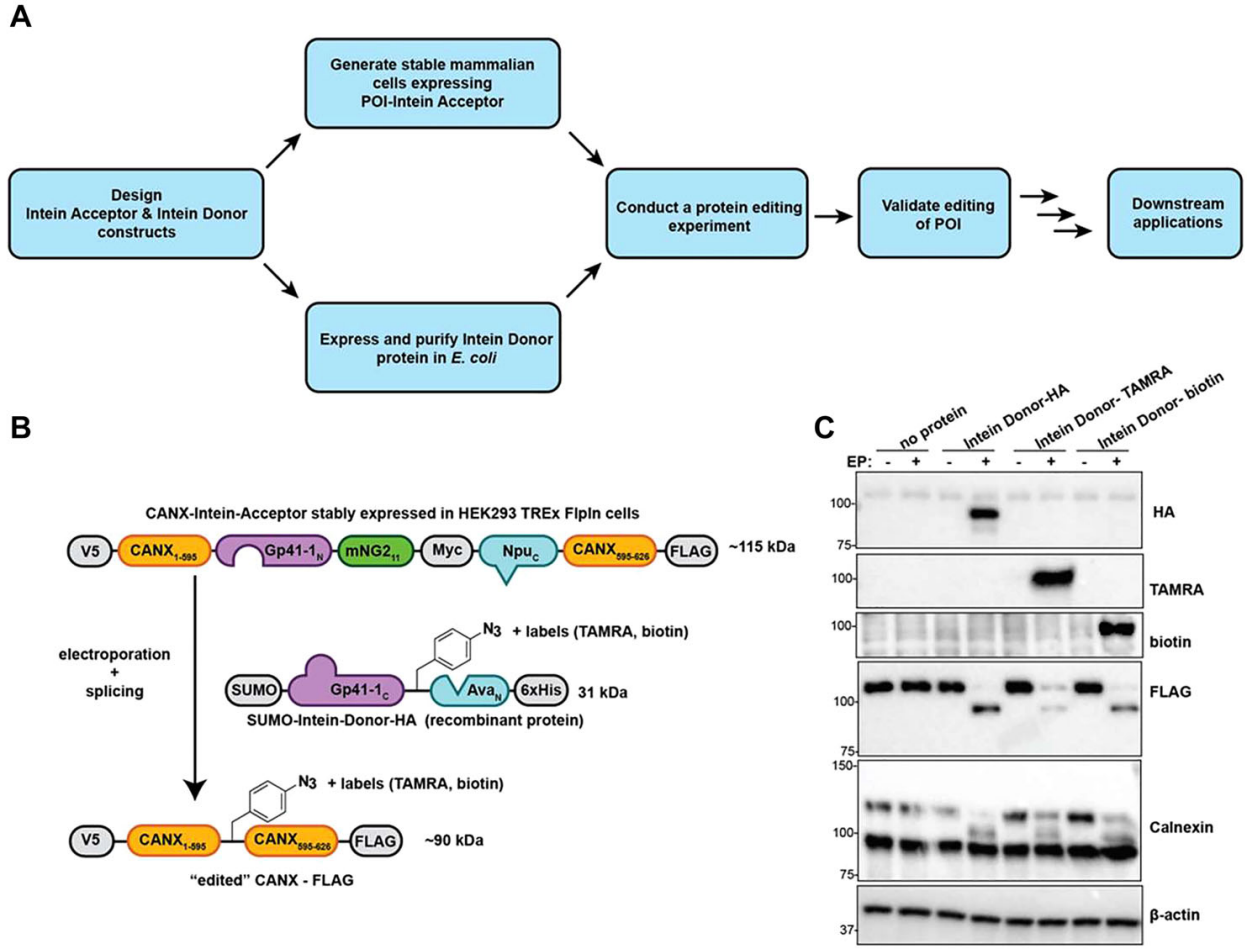
Workflow and potential outcomes for conducting a protein editing experiment. **(A)** A flowchart for designing a protein editing experiment. **(B)** A cartoon schematic for protein editing of calnexin (CANX), where an intein donor sequence containing either a HA tag or ncAA *p*‐azido‐phenylalanine (pAzF) and a conjugated label were edited into CANX. **(C)** Western blot showing the experiment resulting from **(B)**.

In this protocol, we will outline the general approach for setting up a cellular protein editing experiment (Fig. 2a). We emphasize that the planning process for a protein editing experiment, ranging from editing site selection to building in robust controls, is significant, and our current approaches for determining these sites will be detailed later (see Strategic Planning). We use the Gp41‐1 and Ava_N_:Npu_C_ split intein combination for planning, though other reports have shown editing activity with other split intein combinations that may possess catalytic activity, extein requirements, or other properties that are desirable for a given scenario. Lastly, we include methods to install and subsequently label a click chemistry handle, *p*‐azido‐phenylalanine, a non‐canonical amino acid (ncAA) within the intein donor, such that this ncAA and any label can be edited into a protein in live mammalian cells. This approach was used in our prior work (Beyer et al., 2025) and enabled the addition of an epitope tag, biotin, and TAMRA fluorophore labels into the protein calnexin (Fig. 2b and 2c), among numerous other targets. Overall, we anticipate that this protocol should serve as a generalizable roadmap for planning and carrying out a protein editing experiment.

## STRATEGIC PLANNING: CONSTRUCT DESIGN FOR PROTEIN EDITING

To carry out a protein editing experiment, it is first critical to design well‐thought‐out constructs. Two critical components must be designed for a protein editing experiment: the protein of interest (POI)–intein acceptor to be expressed in mammalian cells and the corresponding recombinant intein donor to be delivered by electroporation. To design these, other factors such as split inteins and addition of epitope tags should also be considered, as discussed in the following section.

### Selection of Split Intein Pairs

First, the two pairs of split inteins should be selected. These must be orthogonal, such that the *N‐*split intein domain reacts only with its corresponding *C‐*domain. There are numerous pairs of split inteins, including many that are orthogonal (Pinto et al., 2020). We note that for editing experiments, recent work has utilized Gp41‐1/Ava_N_:Npu_C_^2^, VidaL/Cfa^3^, and Cat/mut‐Cfa^3^. To date, these are the pairs that have been reliably shown to be orthogonal and functional in this context, and as such, are excellent starting points for a new protein editing experiment.

Beyond this, we anticipate that other split inteins could be used for editing in the future. There is a deep body of work surrounding split inteins and protein splicing [reviewed here (Shah & Muir, 2013)], including many split inteins with potentially desirable features for protein editing. There are split inteins that are conditionally inducible (Ventura & Mootz, 2019), either via light (Böcker et al., 2019), drug addition (Gramespacher et al., 2019), or protease (Gramespacher et al., 2017), which could increase temporal resolution for editing. Other split inteins are asymmetrical, in that the relative size of their *N‐* and *C‐*domains is switched (Bachmann & Mootz, 2015; Burton et al., 2020; Thiel et al., 2014), which could offer the potential to modify the size of the intein acceptor construct. Some split inteins have also been further engineered for increased extein tolerance (Stevens et al., 2017) or solubility (Stevens et al., 2016), which could be beneficial for protein editing. Splicing speed, usually reported as a t_1/2_, varies considerably across split inteins and could also be manipulated for in‐cell protein editing (Shah & Muir, 2013). Lastly, many split inteins possess different extein preferences (Shah & Muir, 2013), meaning that the amino acid “scar” left by editing a given protein could be reduced or eliminated using split inteins with exteins that either match the native sequence within a target protein or exhibit notable extein flexibility (Ye et al., 2026).

### Designing the POI‐Intein Acceptor

In the following sections, we will describe the process of designing intein acceptor and intein donor constructs using the AvaN:Npu and Gp41‐1 split intein pairs, as these have worked well in our hands previously. We note that the extein sequences and domains referenced will be specific for these split intein pairs, though other intein pairs and their extein sequences could easily be substituted as needed. As detailed above, the POI–intein acceptor construct requires extein residues to promote splicing (see Fig. 1). Of these extein residues, the required catalytic residues (Ser and Cys, in this case) are most critical, as splicing will not occur without them. Other extein residues can influence the rate of splicing, making these important but not always essential, depending on the residue and the experiment. We have previously shown that the Gp41‐1 and Ava_N_:Npu_C_ pairing can be very flexible for extein residues, in that only the catalytic residues and the *C‐*extein “FN” for Ava_N_:Npu_C_ appear to be absolutely required (Beyer et al., 2025).

Additionally, it is crucial to determine what sequences should be contained between the split intein domains in the intein acceptor. In our group, it has been standard to include an epitope tag for assessing splicing, as well as a fragment of a split fluorescent protein, mNeonGreen_11_, for clear visualization of intein acceptor–tagged proteins using microscopy. This combination of an epitope tag and mNeonGreen_11_ has proved successful across 5+ edited proteins (Beyer et al., 2025) and serves as a general intein acceptor in our group. However, it is conceivable that other studies may require other sequences within the intein acceptor, such as conserved regions for protein targeting or function. We believe it is likely that many other sequences will be conducive for protein editing; however, at present, it remains untested. We attempted to use a complete mClover3 in previous work, which unfortunately resulted in incomplete splicing. This indicates that the contents of the intein acceptor do have implications for splicing, and again we emphasize that this relationship between the intein acceptor and splicing ability is not well understood at present.

It is critical to first identify the purpose of the editing experiment with respect to the POI. The purpose determines the approach to identifying potential editing sites. To date, we have identified two larger purposes:

1. To label a POI internally (i.e., target residues to edit are not specified)
2. To manipulate specific residues (i.e., target residues are specified)

These purposes will require different approaches to experiment design. It is recommended that new users read both sections and understand the priorities of both approaches, as well as the trade‐offs required.

#### 1. To label a POI internally (i.e., target residues to edit are not specified)

This approach allows for the identification of the most optimal editing sites in a protein of interest, to the best of our current knowledge.

(A) Examine the sequence of the POI. First, identify places where a Ser and then a Cys occur nearby. Further, search for places with multiple Ser residues or a Cys‐Phe‐Asn, if these extein sequences (or sequences close to these) occur naturally in the protein.
(B) Examine the structure of the POI. If experimental structures exist, use these first. If the POI lacks structural data, use a structure prediction program such as AlphaFold3 (Abramson et al., 2024) to predict the structure. To date, we have focused editing efforts such that the two splicing events will occur in loops, in hopes that the local flexibility will aid splicing and mitigate structural perturbations to the edited protein. Compare the regions identified in (A) to the structure and identify splicing sites that occur within loops. It may be possible to position the two splicing junctions within loops while editing in a longer β‐strand.
(C) Add epitope tags that will aid in validating splicing. It is standard to include an epitope tag between the split inteins within the acceptor (such that disappearance of this tag indicates editing) and an *N‐* or *C‐*terminal epitope tag on the POI to monitor overall POI size throughout an editing experiment.

#### Pros and cons of labeling a protein with no preference for site

Pros: Likely to require fewer mutations and ability to target domains or regions that are unlikely to influence the POI's characteristics

Cons: Unable to probe specific areas of interest within a POI

#### 2. Labeling a protein with specific residue(s) targeted for editing

(A) Identify the residue(s) that are of interest for editing (for example, sites of post‐translational modifications)
(B) Examine the sequence of the POI. Search the region surrounding the residue of interest for upstream residues that resemble the GY‐SSS and then a further downstream CFN motif, as required for splicing. It can be useful to identify a few candidate sites, if possible.
(C) Examine the structure of the POI. If experimental structures exist, use these first. If the POI lacks structural data, use a structure prediction program such as AlphaFold3 (Abramson et al., 2024) to predict the structure. To date, we have focused editing efforts such that the two splicing events will occur in loops, in hopes that the local flexibility will aid splicing while mitigating structural perturbations to the edited protein. Compare the regions identified in (A) to the structure and identify splicing sites that occur within loops. It may be possible to position the two splicing junctions within loops while editing in a longer β‐strand.
(D) Add epitope tags that will aid in validating splicing. It is standard to include an epitope tag between the split inteins within the acceptor (such that disappearance of this tag indicates editing) and an *N‐* or *C‐*terminal epitope tag on the POI to monitor overall POI size throughout an editing experiment.

#### Pros and cons of labeling a protein as a specific residue

Pros: Precise control over a residue or region of interest

Cons: Higher likelihood that mutations will be required, or that sub‐optimal sequence/structure disruption will be required to access a particular region

### Case Study: Situating an Intein Acceptor in c‐Myc Protein

Here, we will use the transcription factor c‐Myc as an example for designing a protein editing experiment. The objective of the experiment is to investigate the reported short half‐life (20–40 min) of c‐Myc (Gregory & Hann, 2000), and as such we want to position the intein acceptor in a portion of the protein relevant for protein degradation.

It is known that c‐Myc degradation is controlled by a phospho‐degron in the Myc homology box I (MBI) domain, corresponding to residues 45–64 within c‐Myc (Welcker et al., 2004). Therefore, we want to place the intein acceptor within this region.

**Figure 3:**
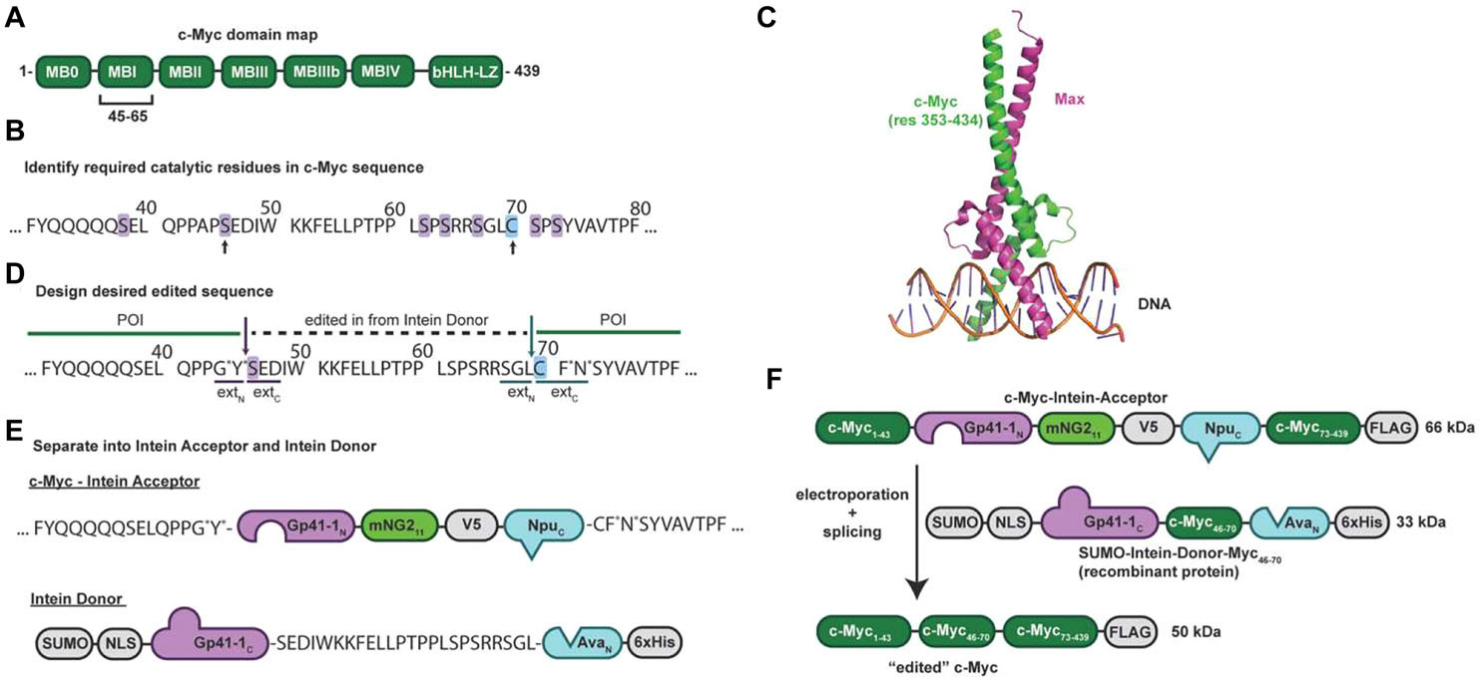
Designing protein editing constructs with c‐Myc as an example. **(A)** A domain map of c‐Myc. **(B)** Identifying potential catalytic residues (purple for serine, blue for cysteine) for splicing in the region of interest in c‐Myc. **(C)** A structure of the ordered, DNA‐binding portion of c‐Myc (PDB: 1NLW). **(D)** Once the catalytic residues have been determined for an editing experiment, map which residues will be part of the intein acceptor and which ones will come from the intein donor. **(E)** Using the map in **(D)**, separate the residues onto intein acceptor and donor constructs and add the required split intein domains and epitope tags. **(F)** Final cartoonized construct map for a c‐Myc protein editing experiment.

First, we examine the sequence of c‐Myc, beginning by identifying the MBI domain (Fig. 3a). Next, we identify the extein residues required for splicing. Gp41‐1 requires a catalytic Ser, and Ava_N_:Npu_C_ requires a Cys. While other extein residues can be critical for splicing efficacy, the Ser and Cys are absolutely required for splicing activity. We can locate a number of Ser and Cys residues in this region (Fig. 3b).

Next, we would typically examine the experimentally determined or computationally predicted model of c‐Myc, where we would identify loops or other flexible regions that appear likely to tolerate splicing. With c‐Myc, there are published structures of the structured DNA‐binding domain (Nair & Burley, 2003) (Fig. 3c). However, c‐Myc also contains significant intrinsically disordered regions, including the MBI domain of interest here. In this case, because this portion of c‐Myc is so disordered, it seems reasonable that almost any area in this region could be amenable to splicing. We emphasize that in most cases, it will be essential to find loops and smaller unstructured regions for splicing sites. To this end, we select Ser46 and Cys70 as the catalytic residues that we will use for splicing, where the intein acceptor will be situated between these residues (Fig. 3d and 3e).

With these established, we can now work to implement the additional extein residues that influence splicing. Gp41‐1 prefers exteins of GY‐SSS, where Ser46 will be the first Ser. To adapt c‐Myc to include the N‐extein residues, we will mutate Ala44 and Pro45 to Gly44 and Tyr45. Next, our previous work has shown that Gp41‐1 will function with a single Ser, so we can retain native c‐Myc residues ED following the catalytic Ser. For the second split intein pair, AvaN:NpuC, exteins of AEY‐CFN are preferred. It is known that the N‐extein AEY is not essential and has little impact on splicing, so we can retain native c‐Myc residues SGL. However, the CFN C‐extein is essential for splicing, so Ser71 and Pro72 will be mutated to Phe71 and Asn72. Together, this results in 4‐point mutations that will be carried through to edited c‐Myc (Fig. 3f). We encourage users to test the impact of even fewer mutations if time and resources allow. Here, we have demonstrated our current approach to designing a protein editing experiment and situating the intein acceptor construct, for a given protein c‐Myc. We anticipate that these principles can be readily extrapolated to other diverse proteins.

### Designing the Intein Donor

The intein donor construct contains the amino acid sequence that will be “edited” into the POI. We have shown that this “cargo” sequence can range in size from a few residues to entire protein domains. This cargo can also include non‐canonical amino acids (ncAAs) installed into the intein donor in *E. coli*, which could include authentic post‐translational modifications, click chemistry handles, photo‐crosslinkers, and so on. The contents of the intein donor, rather obviously, are up to the user and will depend on the downstream experiments desired. Again, while the limits of the intein donor contents have not been robustly determined, we anticipate that there is more flexibility here than in the intein acceptor. Additionally, we emphasize that it is possible to design several intein donors for a given intein acceptor and vice versa. This could be useful for modelling different mutations in one region, or for installing labels at multiple sites in one region. Additionally, implementing a click chemistry handle such as *p*‐azido‐phenylalanine (pAzF) in the intein donor can be useful for rapidly generating intein donors with diverse functionality by clicking on various labels. In this protocol, we will include methods to install pAzF, as it enables the addition of many diverse labels, has a robust genetic code expansion (GCE) system (Miyake‐Stoner et al., 2009), and is widely available commercially.

From a technical perspective, the intein donor construct should be cloned into a pET28 vector or similar for high levels of expression in bacteria. We have demonstrated that the intein donor protein, at least one utilizing Gp41‐1/Ava_N_:Npu_C_, is extremely prone to insolubility (Beyer et al., 2025), and we expect that it will remain so with most cargos. As such, we have developed an expression and purification workflow to successfully isolate insoluble intein donor protein.

### Addition of Epitope Tags to Validate Editing

Lastly, it is imperative that protein editing can be validated. To do this, our group regularly employs Western blotting for preliminary studies. To enable straightforward verification, we use common epitope tags (FLAG, HA, V5, Myc, 6×His, etc) positioned throughout the constructs. An epitope at the *N‐* or *C‐*terminus of the target protein allows us to see the size of the protein pre‐ and post‐editing. An epitope within the intein acceptor should be detectable prior to editing, but it should then move to a very low molecular weight or “disappear” following editing. A 6×His tag located at the *C‐*terminus of the intein donor is essential for purification of the intein donor, and it serves as an important indicator of splicing; this epitope should be detectable inside cells (corresponding to the delivered intein donor) but should not increase in molecular weight, as both split intein pairs should splice to yield a very small 6×His‐tagged intein domain. A higher molecular weight 6×His signal is indicative of the second intein not successfully splicing.

**Figure 4:**
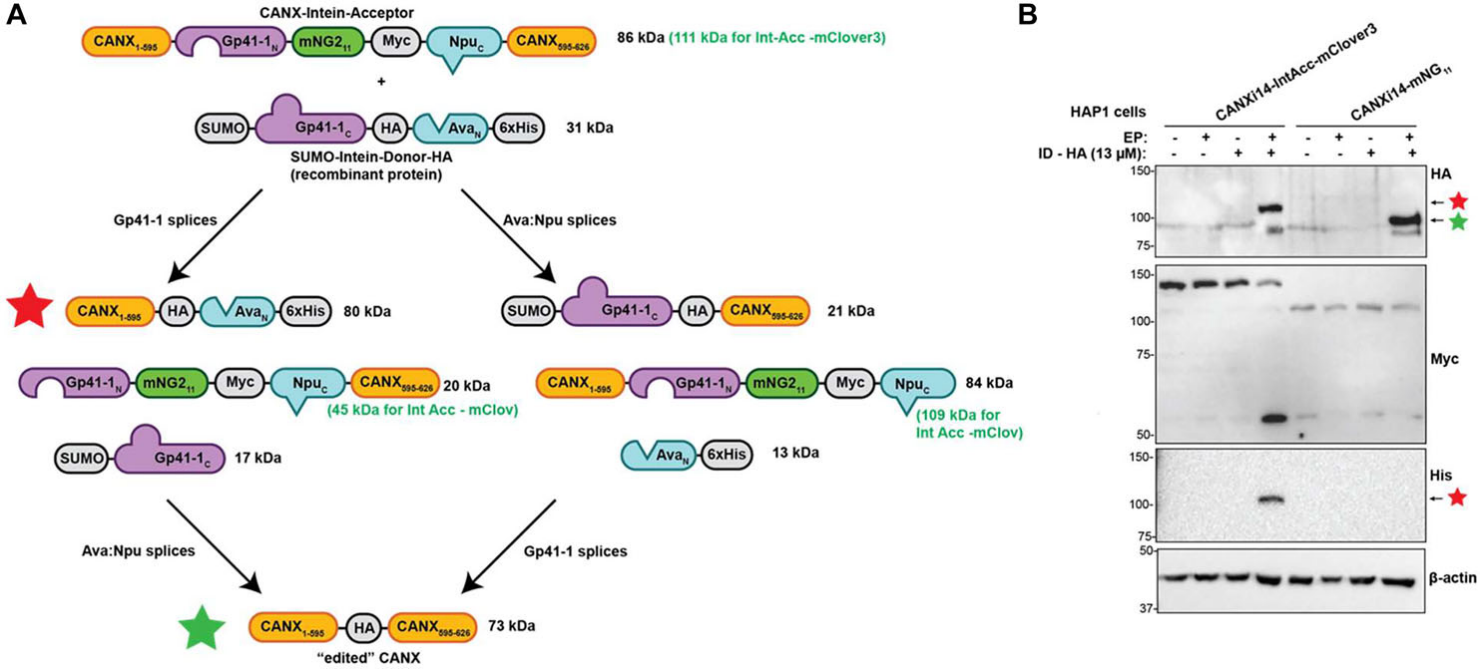
Incomplete splicing is easily detected by added epitope tags. **(A)** Protein editing requires splicing of two inteins. Either splicing event theoretically could happen first, so there are two “routes” to the final spliced product. Possible intermediates are shown here, with a key off‐target intermediate denoted with a red star. The properly edited product is denoted with a green star. **(B)** Western blots resulting from a protein editing experiment using these two different intein acceptors. The undesired intermediate product is again denoted with a red star, while the editing product is shown with a green star.

As an example, we previously tested intein acceptors where the split intein domains were separated by either a full mClover3 or a small mNeonGreen2_11_ fragment (Beyer et al., 2025) (Fig. 4a). We used Western blotting and epitope tags for assay splicing (Fig. 4b). Using the added epitope tags, particularly the 6×His on the intein donor, it is obvious that the intein acceptor with mClover3 is not splicing fully, instead stopping at an undesired intermediate.

### Strategic Planning: Experiment Design

Proper controls are imperative for protein editing experiments, as there are numerous variables and possible outcomes. This is a new and developing technology, and robust controls are essential for validating results.

**Figure 5:**
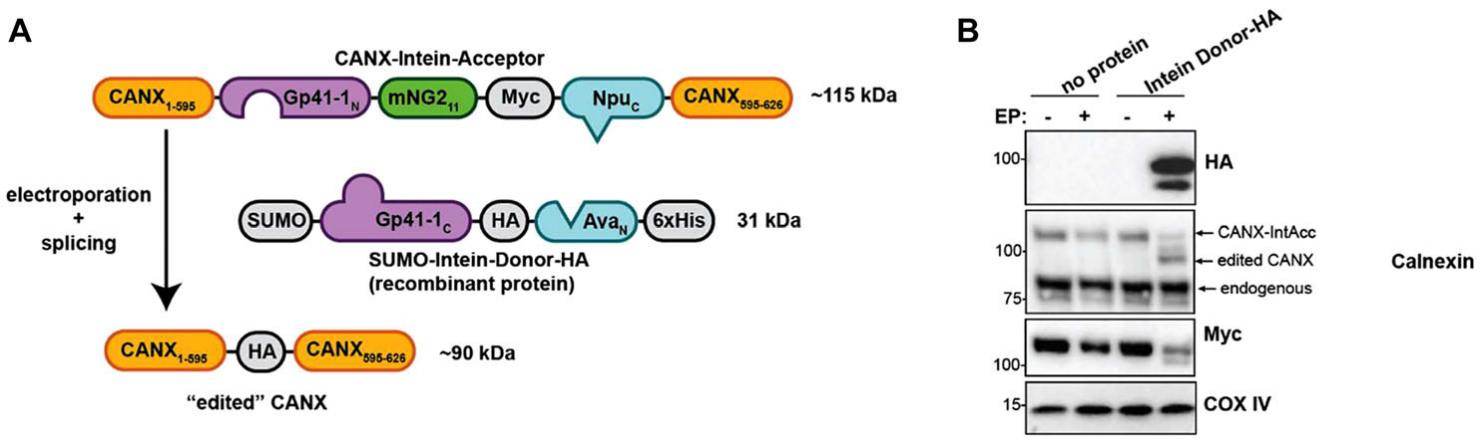
Basic protein editing experiment with appropriate controls. **(A)** A cartoon schematic showing the editing of a HA tag into calnexin (CANX). **(B)** Western blot resulting from the experiment in **(A)**.

First, appropriate controls are required during electroporation, if used for protein delivery (other controls may be required for other protein delivery methods, e.g., lipid nanoparticles). It is standard to include a matrix of conditions that are with/without protein and with/without electroporation, as demonstrated in Fig. 5. It is essential that there is no splicing without intein donor delivery via electroporation and, similarly, no uptake of intein donor without electroporation (Fig. 5a and 5b). It is also possible to create a catalytically inactive intein donor that is not capable of splicing by mutating several essential residues to alanine. Using the combination of Gp41‐1 and Ava_N_:Npu_C_, we used mutations C1A, N37A, and S+1A in the intein donor construct (Beyer et al., 2025). These are useful negative controls.

Further, it is critical to ensure that a POI is being edited as anticipated. In our group, it is standard to validate splicing by Western blotting for the edited protein and epitope tags. As discussed at length previously, building epitope tags into a protein editing experiment will be advantageous for characterizing splicing. It is critical that epitope tags are positioned appropriately such that splicing can be tracked. We strongly recommend mapping out the edited protein sequence with the anticipated molecular weight, as well as for any intermediates resulting from incomplete splicing from either split intein. It is useful to have these mapped constructs and masses established to analyze data resulting from Western blots. To aid in this, we have developed a web tool, which can be found at www.burslemlab.com/proteinediting. Lastly, to most definitively characterize splicing, we recommend that the edited POI be isolated from cells, using a biotin moiety or an epitope tag, and then subjected to bottom‐up proteomics to confirm the amino acid sequence, particularly the peptides(s) that bridge the two splice sites.

*NOTE*: All protocols involving animals must be reviewed and approved by the appropriate Animal Care and Use Committee and must follow regulations for the care and use of laboratory animals. Appropriate informed consent is necessary for obtaining and use of human study material.

## Basic Protocol 1 EXPRESSION OF INTEIN DONOR CONTAINING pAzF

Basic Protocols 1 and 2 are written for an ncAA (pAzF) to be installed into the intein donor. pAzF and the corresponding alkyne or DBCO labels are widely available commercially, and labeling strategies are well worked out. We emphasize that intein donors do not need to contain ncAAs, and/or can instead employ other ncAAs beyond pAzF. If using other ncAAs, it is recommended to follow specific guidelines and specifications for the given ncAA, such as using the appropriate machinery vectors and cell lines for GCE.

### Materials

BL21(DE3) chemically competent cells (Invitrogen)
Relevant plasmids
  pET28 for intein donor expression
  pDule2 GCE machinery (for pAzF incorporation)
SOC (see recipe)
LB agar (see recipe)
LB plate
Relevant antibiotics (Kan for intein donor; Spec or other for GCE machinery)
IPTG, IM
ncAA (pAzF; Sigma‐Aldrich)
MQ H_2_O
NaOH, 8M

Petri dishes, 10 cm
Conical tubes, 50 mL
Heating block or water bath maintained at 42°C
Eppendorf tubes, 1.7 mL
Orbital shaking incubator capable of 250 rpm and kept at 37 °C
Conical centrifuge tubes, 15 mL
Centrifuge capable of 4500 rpm and compatible bottles
Serological pipettes
Pipette gun
Baffled flask, 2 L
Spectrophotometer capable of measuring OD600

#### Day 1: Transformation

1 Place relevant plasmid stocks on ice. Obtain a 50 µL aliquot of chemically competent BL21(DE3) cells and quickly thaw in hand before placing on ice.
2 Add 2 µL of the plasmid required to the aliquot of competent cells and mix by gently pipetting. Ensure cells are collected at the bottom of the tube.
3 Allow the cell and plasmid mixture to rest on ice for 30 min. Set the water bath or heating block to heat to 42°C.
4 Place the tube containing cells and plasmid on the heating block for exactly 45 s.
5 Return cells to ice for exactly 2 min.
6 Gently add 450 µL SOC to cells.
7 Place the Eppendorf tubes into the shaking incubator at 37 °C and allow to recover for 1 hour.
8 In the meantime, obtain the LB agar plate with appropriate antibiotics (kanamycin and spectinomycin).
9 Following the 1‐h recovery, plate 100 µL cells onto the LB agar plate using a glass spreader, with a Bunsen burner to maintain a sterile environment.
10 Allow plates to dry by leaving them on the bench, with the lid cracked open, for 10–15 min.
11 Close the plates, invert, and place them in the 37 °C incubator to allow colonies to grow.

#### Day 2: Prepare overnight starters

12 Add 5 mL LB to a 15‐mL centrifuge tube. Add kanamycin and spectinomycin. If eventually planning a 1‐L culture, make 2 × 5 mL starters.
13 Use a sterile pipette tip to touch 10–20 colonies from the plate and eject the pipette into the starter culture.
14 Move starters to the incubator to grow at 37 °C and at least 200 rpm.
15 Prepare reagents for the next day:
  a. 1 L LB; autoclave
  b. 1M IPTG

#### Day 3: Express intein donor

16 Add kanamycin and spectinomycin to the 1‐L culture.
17 Inoculate the 1‐L culture with 10 mL of overnight starter (1% of larger volume).
18 Grow at 37 °C at ∼200 rpm.
19 Begin taking OD measurements of the culture 2 h after inoculation, until the OD is between 0.4 and 0.6.
20 At this time, remove culture from the incubator.
21 Prepare the ncAA solution (here, 4 mL of 250 mM pAzF):
  a. Weigh out 206.2 mg of pAzF and add to a 15‐mL conical tube.
  b. Add 3.75 mL of MQ H_2_O.
  c. To dissolve pAzF, add 0.25 mL of 8M NaOH (2 molar equivalents to the ncAA)
  d. Vortex until ncAA is dissolved.
  e. Note: If using a different ncAA, refer to appropriate literature for that ncAA to determine solubility and stability.
22 Add pAzF to the culture such that the final concentration is 1 mM. If not using pAzF, refer to relevant literature to identify standard concentrations of ncAA in cultures.
23 Add IPTG to the culture to induce intein donor expression, such that the final concentration is 1 mM. If not using the pET28 vector, ensure that the intein donor will be expressed with relevant inducers.
24 Return culture to the 37 °C incubator and continue growth overnight, such that the protein is expressed between 18 and 24 h.
  Note: Our lab has shown that expression temperature impacts expression levels of the intein donor (Beyer et al., 2025). We observe that a low expression temperature (16 °C) yields extremely little protein. A moderate temperature (25 °C) yields more protein in the soluble fraction, but less protein overall. Expressing at 37 °C gives the highest yields of protein, but this protein is largely sequestered in the insoluble fraction. In general, we express intein donor constructs at 37 °C, with the expectation that the majority of the intein donor will be insoluble in the pellet. It has become standard in our lab to pool the resolubilized protein from the pellet with the soluble fraction to maximize our yields. This approach also allows for variability in solubility with different cargos, though in general we observe that intein donors with varying cargos are still prone to insolubility.

#### Day 4: Harvest cells

25 Transfer culture to 450‐mL large centrifuge bottles and balance before centrifuging for 20–30 min, 4°C, 12000 rpm.
26 Pour off all but ∼15 mL medium and resuspend pellet using this medium with a serological pipette and pipette gun. Transfer to a 50‐mL tube and centrifuge again (20 min, 4°C, 4500 rpm).
27 Pour off all the medium and place the pellet in a −80°C freezer.

## Basic Protocol 2 PURIFICATION AND LABELING OF RECOMBINANT INTEIN DONOR

### Materials

Frozen *E. Coli* cell pellet (from Basic Protocol 1)
Lysis buffer (see recipe)
Protease inhibitor tablets
Resolubilization buffer
Dialysis buffer
NiNTA resin (Qiagen)
EtOH
Milli‐Q H_2_O
Wash buffer
Elution buffer
Sizing buffer
Glycerol
Label‐DBCO (e.g. biotin‐DBCO, TAMRA‐DBCO)
Liquid N_2_

Serological pipettes, 10 mL
Probe sonicator
High‐speed centrifuge capable of 13,000 rpm (29,097 × g)
Oakridge tubes
Falcon tubes, 50 mL
Rocker or rotator (room temp. and 4°C is ideal)
Beaker, 2 L
Magnetic stir bar
Magnetic stir plate
Dialysis tubing, 10 kDa
Conical tubes, 50 mL
Gravity column
SDS‐PAGE
Fast protein liquid chromatography (FPLC) and appropriate size exclusion chromatography (SEC) column (e.g. Cytiva S200 column) equipped with a UV detector
Fraction collector
10‐kDa spin concentrator tubes, 15 mL (Amicon)
Benchtop centrifuge capable of 4500 rpm (4347 × g)
Conical centrifuge tubes, 15 mL
Bicinchoninic acid (BCA) assay
Eppendorf tubes, 1.7 mL

#### Prior to starting

1 Prepare the following buffers (see recipe):
  a. Lysis buffer
  b. Resolubilization buffer
  c. Sizing buffer

#### Day 1: Isolate the insoluble fraction containing the intein donor protein

2 Retrieve the frozen harvested *E. coli* cell pellet and place it on ice.
3 Add ∼20 mL of lysis buffer to the pellet for every 1L of expression culture yielding the pellet, ensuring that protease inhibitors have been added as appropriate.
4 Allow the pellet (with added lysis buffer + protease) to thaw for ∼30 min until the pellet can be resuspended in the lysis buffer by vortexing. Remove any cell clumps by pipetting up and down with a 10‐mL serological pipette.
5 Lyse the cells using probe sonication on ice (50% power; 4 s on, 2 s off; for a total time of 15 min). Other lysis methods are anticipated to be suitable as well. Take care to keep the lysate cold and on ice as much as possible.
6 Clear the lysate using high‐speed centrifugation. Transfer cell lysate to an Oakridge tube and clear the lysate using high‐speed centrifugation (e.g., 13,000 rpm, 4 °C, 30 minutes).
7 Retain the resulting supernatant in a 50‐mL Falcon tube and store at 4 °C. This supernatant will contain already solubilized intein donor.
8 Add ∼30 mL resolubilization buffer to the pellet in the Oakridge tube. Pipette up and down with a serological pipette to aid resuspension. The pellet may remain in some small clumps.
9 Rock or rotate the resolubilized pellet overnight at 4 °C.

#### Day 2: Solubilize intein donor protein

10 Prepare 2 L of dialysis buffer. Store in a 2‐L or larger beaker at 4 °C, with a magnetic stir bar added.
11 Clear the resolubilized pellet solution in a high‐speed centrifuge (e.g., 13,000 rpm, 4 °C, 30 min). The intein donor protein is now in the supernatant.
12 Transfer the supernatant from the Oakridge tube to a 10‐kDa dialysis tubing (or another adequately sized dialysis tubing) and secure with dialysis clips.
13 Submerge the dialysis tubing containing the protein into the dialysis buffer and secure with a float or to the side of the beaker.
14 The dialysis setup should be kept at 4 °C overnight, using the stir bar to mix the buffer in the beaker.

#### Day 3: Purification of intein donor by NiNTA gravity column

15 Transfer protein from dialysis tubing to a 50‐mL conical tube.
16 Set up a gravity column. Choose one of appropriate size to hold the volume of both the initially soluble supernatant and the resolubilized supernatant.
17 Use a serological pipette to add 50% NiNTA resin in EtOH slurry to the column. For 1L of culture, use ∼ 8 mL of NiNTA:EtOH slurry for a bed volume (BV) of 4 mL.
18 Allow the storage EtOH to run through the column.
19 Wash the resin by gently adding 2 × 10 BV of Milli‐Q H_2_O.
20 Equilibrate the resin by gently adding 2 × 10 BV of lysis buffer.
21 Cap the lower end of the column securely. Add the original soluble supernatant from Day 1 and the freshly dialyzed resolubilized supernatant to the column. Then cap the top of the column securely.
22 Incubate the column by rocking or rotating at 4 °C for an hour, or overnight if need be.
23 Retrieve the column and allow resin to settle to the bottom.
24 Place a 50‐mL conical tube underneath the column. Remove the bottom cap and collect the resin flow‐through fraction.
25 When the resin has run dry, gently add 5 BV of wash buffer to the column without disturbing the resin. Collect this wash after passing through the column.
26 Gently wash the column with 5 BV of wash buffer again. Collect this wash fraction in a separate tube.
27 Once the resin has run dry, add 15 mL of elution buffer to the column. Securely cap both ends of the column and rock at room temperature for 15 min.
28 Collect the eluent in a 15‐mL conical tube.
29 Add another 15 mL of elution buffer to the column and rock at room temperature again. This will elute any remaining protein.
30 Collect this second eluent in another 15‐mL conical tube.
31 Add water to the column and wash NiNTA resin as appropriate.
32 Run an SDS‐PAGE gel with samples from the retained resin flow‐through, washes, and eluents to determine which fractions contain purified intein donor.
33 Pool these samples into a 50‐mL conical tube. In our hands, often the second wash and both eluents will be pooled together.
34 Store the pooled protein on ice at 4°C overnight. If pAzF has been incorporated, cover the protein with foil as pAzF is light sensitive.
35 Prepare for the size exclusion chromatography using FPLC the following day:
  a. Prepare 1L of sizing buffer.
  b. Equilibrate the SEC column and FPLC with 2 × column volume (CV) of water and then 2 CV of sizing buffer. The sizing buffer contains glycerol; be sure to decrease the flow rate to avoid damage to the column and instrument.
  c. Load a fraction collector with appropriately sized tubes, if using.

#### Day 4: Purification of intein donor by size exclusion chromatography and labeling

**Figure 6:**
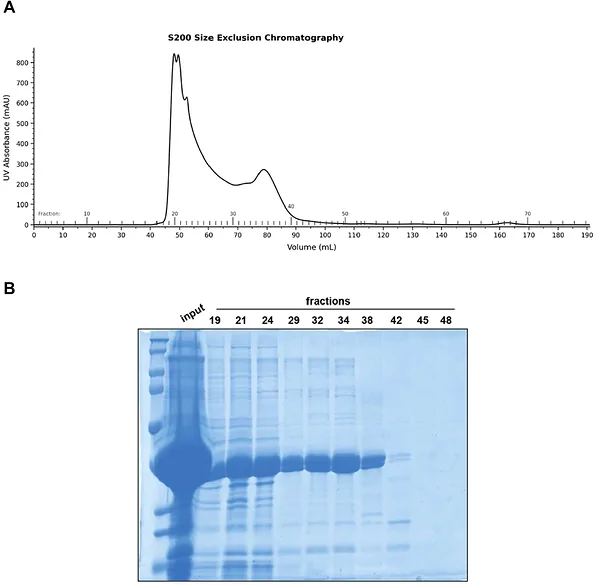
Size exclusion chromatography (SEC) purification of intein donor. **(A)** The UV trace for an intein donor without pAzF is shown. Note that if the intein donor does contain pAzF, it is best to disable the UV detector. **(B)** Fractions from the SEC purification run on an SDS‐PAGE gel. Here, fractions 19–42 were pooled.

36 Concentrate the pooled fractions using a 15‐mL 10‐kDa molecular weight cutoff spin concentrator tube until the protein is at a volume such that it can be loaded onto the FPLC instrument. In general, centrifuge at intervals of 30 min, 4 °C, and 4347 × g.
37 Prepare FPLC to begin a SEC purification.
38 Centrifuge the protein sample to clear any debris (21,000 rcf, 4 °C, 5 min).
39 Load protein sample onto the FPLC instrument as appropriate and then continue the purification method.
40 Collect fractions as appropriate on the instrument. A UV detector can be used to identify when the protein comes off the column (Fig. 6a); however, if the intein donor contains pAzF, turn off the UV detector in the FPLC and plan to check fractions exclusively using SDS‐PAGE.
  Note: In our hands, the intein donor protein tends to elute from the column immediately after the void‐volume UV peak. The intein donor runs as one peak followed by a highly extended, broad peak. We have not observed any functional difference between the protein from these distinct peaks and believe that this phenomenon is caused by the insolubility of the protein.

**Figure 7:**
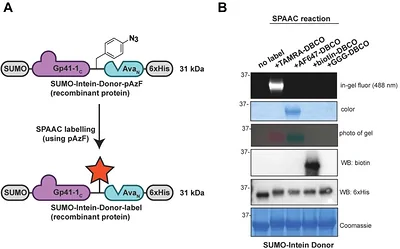
Labeling recombinant intein donor via click chemistry. **(A)** The pAzF ncAA reacts with a label‐DBCO moiety for a strain‐promoted azide–alkyne cycloaddition (SPAAC) reaction, resulting in the labeling of the intein donor. **(B)** Western blot and in‐gel fluorescence qualitatively confirm the labeling of the intein donor with various labels.

41 Check fractions by SDS‐PAGE to determine which ones contain pure intein donor. Pool these fractions. See Fig. 6b for an example.
42 Determine the concentration of the pooled intein donor using a BCA Assay. Determine overall yield of pure protein from the expression. We routinely observe yields between 10–30 mg pure protein per liter culture.
43 Next, for labeling, convert the protein concentration from mg/mL to µM.
44 Obtain the label as a stock solution. Add 5 molar equivalents of label to the protein (for example, if the protein is 10 µM, the label should be at 50 µM in the reaction).
45 Allow the protein and label to rock overnight (at least 16 h) at room temperature. In our experience, the purified intein donor is stable through this treatment.
46 Concentrate the labeled protein using 10‐kDa molecular weight cutoff spin concentrator tubes.
  a. First, wash excess label away. This can be done by concentrating down to a low volume (∼ 1 mL) and filling the concentrator tube with sizing buffer. Repeat 2–3 times or until the flow‐through is clear, if using a fluorescent label.
  b. Obtain the desired concentration, checking through a BCA assay.
47 Aliquot into tubes, label, and flash‐freeze in liquid N_2_. Store at −80 °C and avoid freeze–thaws of the protein.
48 Use SDS‐PAGE gels, in‐gel fluorescence, Western blots, mass spectrometry, or other suitable methods to confirm complete labeling (Fig. 7).
  Note: Here, we use strain‐promoted azide‐alkyne cycloaddition (SPAAC) to label the pAzF‐containing intein donor. CuAAC is also possible on the purified protein; we recommend using TBTA as the catalyst to avoid protein precipitation.

## Basic Protocol 3 PREPARATION OF MAMMALIAN CELLS FOR EDITING EXPERIMENTS

### Materials

FlpIn HEK293 TREx cells (Thermo Fisher Scientific)
Hygromycin B
Complete DMEM
Transfection reagent (PolyJet, PEImax, or similar)
pcDNA5‐POI‐intein acceptor
pOG44
DPBS
Trypsin‐EDTA, 0.25%
Tetracycline

6‐well dishes
T25 flasks
T75 flasks
Cryostock tubes
Mr. Frosty freezing container
Liquid nitrogen dewar
Biosafety cabinet
Cell culture incubator (37 °C, 5% CO_2_)
Slow‐freezing container for cryostocks

1 Thaw a cryostock of the HEK293 TREx cells and expand until cells are recovered from freezing and doubling as expected.
2 To determine the appropriate concentration of hygromycin B to use for selection, conduct a kill curve experiment. Plate the cells into a 6‐well dish and add variable amounts of hygromycin B (e.g., a series from 0–1 mg/mL). Monitor cells for 10–14 days. For future selections, use the lowest concentration of hygromycin B that killed all cells in a well.
3 Plate cells into a 6‐well dish in complete DMEM, such that they are 80% confluent on the day of transfection. Individual T25 flasks can also be used.
4 Following the transfection reagent's instructions, transfect pOG44 and pcDNA5 in a 9:1 ratio (for a typical well in a 6‐well dish, 900 ng pOG44 to 100 ng pcDNA5.
5 The next morning, refresh the medium with complete DMEM.
6 At 48 h after transfection, refresh the medium and add hygromycin B to the concentration determined in the kill curve experiment above (we routinely use 50 µg/mL).
7 Continue refreshing the medium and hygromycin B for 10–14 days, or until control transfected cells have died.
8 Vacuum off the medium, wash with DPBS, and vacuum off the DPBS.
9 Add 200 µL 0.25% trypsin‐EDTA and dislodge cells by tapping.
10 Add 800 µL of complete DMEM and transfer cells to a new T25 flask. Add an additional 4 mL of complete medium. Do not add hygromycin B.
11 Let cells recover in complete DMEM and begin expanding. Cells may be very sparse. Continue to refresh medium every few days until cells are ready to be split. Expand cells into larger T75 flasks.
12 While expanding cells, verify that the selected cell population expresses the POI–intein acceptor. Treat cells with 1 µg/mL tetracycline to induce gene expression. Use a Western blot or another assay to verify POI–intein acceptor prior to further experiments.
13 Freeze aliquots of the generated cell line in cryostock tubes. Freeze gradually in the freezing container at −80 °C for 24–48 h before moving to a liquid nitrogen dewar for long‐term storage.

## Basic Protocol 4 PROTEIN EDITING VIA ELECTROPORATION

### Materials

Cell line cryostocks (from Basic Protocol 3)
Tetracycline
Antibiotic‐free DMEM (afDEEM; 10% FBS)
DPBS
Trypsin‐EDTA, 0.25%
Complete DMEM (10% FBS, 1% Pen‐Strep)
Neon electroporation buffers E2 and R (Thermo Fisher Scientific; optional; can instead use a homemade electroporation buffer; see recipe)
RIPA buffer (prepared with protease inhibitor)

6‐well dishes, tissue culture treated
T75 flasks
T125 flasks
Cell culture incubator
Sterile Eppendorf tubes, 1.7 mL
Biosafety cabinet
Pipettes
Conical tubes, 50 mL
Centrifuge capable of 0.1 × g (located in a cell culture room or otherwise extremely carefully cleaned with 70% ethanol)
Cell counter (optional)
Neon NxT Electroporation System 1‐Channel Tubes (Thermo Fisher Scientific)
Neon or Neon NxT electroporation unit (Thermo Fisher Scientific)
Neon electroporation pipette tips, 100 µL (Thermo Fisher Scientific)
Probe sonicator (optional, depends on harvesting method)

#### Strategic planning: Electroporation calculations

Before a protein editing experiment, plan the experiment and conditions thoroughly. It is important to plan controls. For a basic protein editing experiment, 4 conditions are needed (see Fig. 5B). Once the required conditions are planned, calculate the required amounts of cells and protein.

For each “+ protein” condition, 225 µL of cell–protein solution is required; 225 µL is enough for a 100 µL electroporation, and 100 µL of solution is to be plated as a “− EP” control. In general, we use a concentration of 50 µM protein in the cell–protein solution. This can be altered according to specific experimental needs, and we have performed editing with 13–100 µM protein in the solution.

#### 225 µL cell–protein solution

**Table:** 

Cells	90 µL
Protein	_ µL (to achieve 50 µM)
Buffer R	_ µL (to 225 µL)
**Total**	**225 µL**

#### Prior to experiment

1 Thaw a cryostock of the cell line generated in Basic Protocol 3 and expand such that cells recover from freezing and double as expected.
2 Expand cells to T75 or T175 flasks. See note in step 3 for scaling details.
3 The day prior to electroporation, refresh the medium. Add tetracycline to a final concentration of 1 µg/mL in the flasks to induce POI–intein acceptor expression.
  Note: For a basic electroporation experiment, with 4 conditions (Fig. 5B), plan to have at least two T75 flasks 80% confluent on the day of electroporation. This will provide more than enough cells. For larger experiments, scale cells up into T175 flasks to have sufficient material. It is critical to have more cells than needed, in case of electroporation issues (e.g., arcing) where samples would need to be prepared again.

#### Day 1: Electroporation

4 Pipette 1.5 mL of afDMEM into the wells of a 6‐well dish, and place in the incubator to pre‐warm. For other wells/plates, add a generous volume of afDMEM.
5 For each condition, add 750 µL DPBS into a labeled sterile Eppendorf tube. Place in the cell culture incubator to pre‐warm.
6 Obtain cells in a T75 flask or similar from the incubator and move into the biosafety cabinet. Wash cells with 10 mL DPBS and then remove the DPBS by vacuum. Add 2 mL of 0.25% trypsin‐EDTA to the flask and rotate the flask to distribute trypsin. Cells can be detached by gently tapping the flask or by incubating at 37°C for 5 min.
7 Add 8 mL of complete DMEM to the flask and pipette to distribute cells. Then pipette up the entire 10 mL of cells (alternately, pipette 9.5 mL of cells and add back 9.5 mL complete DMEM to maintain the cell line) and transfer to a 50‐mL conical tube.
8 Centrifuge cells for 3 min at 100 × g. After centrifuging, cells should be pelleted to the bottom of the conical tube. Vacuum off the medium, taking care not to dislodge the pellet. Carefully pipette 10 mL DPBS into the tube and gently resuspend cells.
9 Again, centrifuge cells for 3 min at 100 × g. Remove the supernatant by vacuum, leaving the cell pellet in the tube.
10 Resuspend the cell pellet in as little Buffer R as possible, considering the total volume of cells needed for the experiment. Start with that volume of Buffer R.
  Note: If desired, an electroporation buffer (Brees & Fransen, 2014) (see recipe) can be prepared and used in place of Buffer R and Buffer E2.
11 Count the cells resuspended in Buffer R. For a typical slide counter, at least a 10‐fold dilution will be required for an accurate count. Cells should be dense, between 25 and 60 million/mL. Dilute to this range by adding more Buffer R and then counting again, until cells are in the targeted density range.
12 Prepare the protein–cell solutions as outlined in “Strategic planning: Electroporation calculations.”
13 Place an electroporation tube into the pipette stand. Add 3 mL of Buffer E2 to an electroporation tube (2 mL if using Neon NxT).
14 Turn on the electroporation unit and set the electroporation conditions. Our work has shown sufficient delivery to HEK293T and HAP1 cells using 2 pulses of 1400 V for 20 ms. These parameters can be optimized depending on the cell line and application.
15 Remove the Eppendorf tubes containing 750 µL DPBS from the incubator and have them accessible near the electroporator.
16 Carefully load a 100‐µL electroporation pipette tip onto the Neon pipettor. Gently mix the cell–protein solution before taking up 100 µL into the pipette tip.
17 Insert the Neon pipettor into the electroporation tube and verify that it clicks into place.
18 Verify that the sample in the Neon tip does not have any bubbles or air at the top of the tip. Any air bubbles will cause arcing and will damage the sample.
19 Press “Go” on the electroporator and wait until the instrument reads “Complete”. Ensure the electroporation proceeds without arcing. If the apparatus arcs, as indicated by a flash of light near the top of the electroporation tip within the tube, dispose of the sample and the electroporation tip. The tip should not be used again.
20 Carefully pipette the electroporated cell–protein solution into a tube with the pre‐warmed DPBS.
21 Repeat all the remaining electroporation processes to complete the entire experiment.
22 For “− electroporation” control reactions, use a standard, non‐Neon pipette to dispense 100 µL of the cell–protein solution into a prepared tube with pre‐warmed DPBS.
23 Centrifuge all reactions in DPBS for 1 min at 300 rcf. Carefully vacuum off or pipette off the DPBS away from the small cell pellet. Gently resuspend with 750 µL of DPBS.
24 Centrifuge again for 1 min at 300 rcf. Remove the DPBS supernatant as before.
25 Gently resuspend the cell pellet in 500 µL of warmed antibiotic‐free medium. Add to the prepared wells within a 6‐well plate, bringing total well volume to 2 mL.
26 Gently tap or swirl the plate to evenly distribute cells.
27 Place the dish in the incubator and recover. Recovery can range from minutes to hours depending on the purpose of the experiment. A typical recovery time of 4 h allows cells to re‐adhere to the dish before harvesting.

#### Harvest Cells

28 Remove cells from the incubator and place them on ice.
29 Gently add 1 mL DPBS per well. Vacuum DPBS out of the well.
30 Add 30 µL RIPA buffer to each well. Incubate the plate on ice for 10 min.
31 Scrape cells to collect and transfer to an Eppendorf tube. Sonicate the samples to obtain nuclear proteins, if desired. Centrifuge at 17,000 × g for 10 min, 4°C, to pellet debris. Freeze lysate or use in downstream applications immediately.
  Note: Cells can be harvested with a shorter or longer recovery, and using different methods. The methods outlined above are the standard in our lab.

## REAGENTS AND SOLUTIONS

### Dialysis buffer

50 mM HEPES, pH 7.5
300 mM NaCl
5% glycerol
5 mM imidazole

### Electroporation buffer (optional; can be used in place of both buffers provided by Thermo Fisher Scientific) (Brees & Fransen, 2014)

250 mM sucrose
1 mM MgCl2•6H_2_O
Diluted in DPBS

### Elution buffer

50 mM HEPES, pH 7.5
300 mM NaCl
5% glycerol
300 mM imidazole

### Lysis buffer

50 mM HEPES, pH 7.5
300 mM NaCl
5% glycerol

### Resolubilization buffer

50 mM HEPES, pH 7.5
300 mM NaCl
5% glycerol
8M urea

### Sizing buffer

50 mM HEPES, pH 7.5
300 mM NaCl
5% glycerol
(sterile filter sizing buffer)

### SOC (10 mL)

9.8 mL 2×YT (sterile)
100 µL 40% glucose
100 µL 1M MgSO4 (sterile)

### Wash buffer

50 mM HEPES, pH 7.5
300 mM NaCl
5% glycerol
50 mM imidazole

## CONCLUSION

Here, we have presented a protocol for protein editing in mammalian cells. We anticipate that this powerful tool will be widely applicable across a multitude of target proteins, cell lines, and cargos; it should enable precise modifications and manipulations to proteins that were previously out of reach. In summary, we hope that this protein editing technology will enable completely new experiments, with unprecedented resolution, and help deliver new insight into biomolecular processes inside mammalian cells.

### Author Contributions

**Jenna N. Beyer**: Writing—original draft; investigation; visualization; methodology; formal analysis. **Kaitlyn Toy**: Investigation; writing—review and editing; methodology; visualization; software. **George M. Burslem**: Conceptualization; investigation; funding acquisition; writing—review and editing; visualization; methodology; validation; formal analysis; project administration; supervision; resources

### Conflict of Interest

The authors declare no conflict of interest.

## Data Availability

The data that support the findings of this study are available from the corresponding author upon reasonable request.
